# Aberrant Effective Connectivity of the Right Anterior Insula in Primary Insomnia

**DOI:** 10.3389/fneur.2018.00317

**Published:** 2018-05-08

**Authors:** Chao Li, Mengshi Dong, Yi Yin, Kelei Hua, Shishun Fu, Guihua Jiang

**Affiliations:** Department of Medical Imaging, Guangdong Second Provincial General Hospital, Guangzhou, China

**Keywords:** primary insomnia, functional magnetic resonance imaging, effective connectivity, insular cortex, executive function, cognitive impairment

## Abstract

**Objective:**

Daytime cognitive impairment is an essential symptom of primary insomnia (PI). However, the underlying neural substrate remains largely unknown. Many studies have shown that the right anterior insula (rAI) as a key node of salience network (SN) plays a critical role in switching between the executive control network (ECN) and the default mode network (DMN) for better performance of cognitively demanding tasks. Aberrant effective connectivity (directional functional connectivity) of rAI with ECN or DMN may be one reason for daytime cognitive impairment in PI patients. Up to now, no effective connectivity study has been conducted on patients with PI during resting state. Our aim is to investigate the effective connectivity between the rAI and the other voxels in the whole brain in PI.

**Materials and methods:**

Fifty drug-naive patients with PI and forty age- and sex-matched healthy controls were scanned using resting-state functional MRI. Seed-based Granger causality analysis was used to examine effective connectivity between the rAI, including ventral and dorsal part, and the whole brain. The effective connectivity was compared between the two groups and was correlated with clinical characteristics.

**Results:**

Compared with controls, patients showed decreased effective connectivity from the rAI to the bilateral precuneus, the left postcentral gyrus (extending to bilateral precuneus) and the bilateral cerebellum posterior lobe, and decreased effective connectivity from the bilateral orbitofrontal cortex (OFC) to the rAI (single voxel *P* < 0.001, AlphaSim corrected with *P* < 0.01). In addition, effective connectivity from the ventral rAI to the left postcentral gyrus and from the left OFC to the ventral rAI were significantly negatively correlated with Insomnia Severity Index scores (*r* = −0.28/*P* = 0.046 and *r* = −0.29/*P* = 0.038, respectively).

**Conclusion:**

The present study is the first to reveal aberrant effective connectivity between the SN hub (rAI) and the posterior DMN hub (precuneus) as well as decision-making region (OFC) and sensori-motor region in PI. These findings suggest an aberrant salience processing system of the rAI in PI patients.

## Introduction

Primary insomnia (PI) is one of the most common health problems. It is characterized by difficulties in falling asleep, maintaining sleep, or early awakening for at least 1 month (1). The worldwide prevalence of insomnia symptoms is approximately 30–35% and approximately 10% of people are diagnosed with PI (2, 3). Insomnia is associated with cognitive impairment, daytime fatigue, and mood disruption (4, 5). Among a series of adverse consequences caused by insomnia, daytime cognitive impairment is an essential symptom with regard to working memory, episodic memory, and some aspects of executive functioning (4, 6). However, the underlying neural substrate is incompletely understood.

Neuroimaging techniques provided a new avenue to study the pathophysiological mechanisms underlying many psychiatric disorders (7–12). With regard to PI, PET, functional, and structural MR imaging have shown abnormal glucose metabolism (13), activation (14, 15), spontaneous activity (16–18), functional or structural connectivity (19–25), or atrophic structure (26–28) related to the cognitive system, especially the salience network (SN) (11, 21), executive control network (ECN), and default mode network (DMN) (22, 29).

It is worth noting that failed reducing activities of DMN is an important feature of PI during cognitively demanding tasks, such as during working memory task (15). During working memory task, with increasing task difficulty PI patients not only showed reduced activation in task-positive regions but also showed reduced deactivation in task-negative regions (DMN) (15). This study suggested that it was not simply the failure to recruit ECN that was associated with the reduced cognitively demanding task performance, but there was a conjoint failure to deactivate the DMN. However, very little is known about the neural mechanism behind this phenomenon.

The right anterior insula (rAI) as a key node of SN can modulate activity in the ECN and the DMN in healthy individuals for better performance of cognitively demanding tasks (30, 31). It is worth noting that the modulating function of anterior insula is right lateralized. So, we only choose the right side as the seed region but not left insula. Present theory holds that one fundamental mechanism underlying cognitive control is a transient signal from the right fronto-insular cortex, which engages the brain’s attention, working memory, and higher-order control processes while disengaging other systems (such as DMN) that are not immediately task relevant (30, 32). Therefore, aberrant directed functional connectivity (FC) (effective connectivity) of rAI over ECN or DMN may be one reason of daytime cognitive impairment in PI patients. Previous studies have found altered FC in the right insula in PI patients (20–22, 33–35). However, there is no research to study the effective connectivity of the rAI.

In contrast to FC, which is zero time-lagged correlation between time series at spatially distinct regions of brain, the effective connectivity is the time-lagged correlation between time series. Effective connectivity from a region *X* to another region *Y* implies that the neuronal activity in region *X* precedes and predicts the neuronal activity that occurs in region *Y*. As mentioned above, some FC studies have already been performed to study the right insula (including rAI). However, there is still no research to study the directional FC (effective connectivity) in PI. Investigation of effective connectivity in PI patients may deepen our understanding of neurologic mechanism of PI.

We employed Granger causality analysis (36–38) in resting-state functional MR imaging to investigate the rAI-centered effective connectivity. Granger causal influence from a region *X* to another region *Y* implies that the neuronal activity in region *X* precedes and predicts the neuronal activity that occurs in region *Y* (38). Thus, the whole-brain Granger causal analysis is a useful approach to study the effective connectivity that may exist across networks. In contrast to undirected FC which does not support inferences about directed (causal) brain connections, effective connectivity refers to the influence that one neural system exerts over another and quantifies the directed coupling among brain regions. In addition to Granger causal analysis, there are also several ways to capture the directional brain dynamics, such as dynamic causal modeling and structural equation modeling. Granger causal analysis has been widely used to study effective connectivity in normal brains (30), schizophrenia (38), and major depressive disorders (37). To date, to the best of our knowledge, no studies have been published reporting effective connectivity in PI patients. The purpose of this study was to analyze the effective connectivity between the rAI and the whole brain in PI patients using first-order Granger causality analysis and its association with sleep and emotion scales of PI. We hypothesized that the effective connectivity between rAI and ECN or DMN was disrupted.

## Materials and Methods

### Participants

This prospective study was approved by the ethics committee of the Guangdong Second Provincial General Hospital. All PI patients were recruited from the Department of Neurology at Guangdong Second Provincial General Hospital, Guangzhou, China from April 2010 to May 2016. Written informed consent was obtained from all patients. The inclusion criteria for PI patients were (a) all patients must meet Diagnostic and Statistical Manual of Mental Disorders, Fourth Edition (DSM-IV) for diagnosis of PI; (b) patients had been complaining of difficulty falling asleep, maintaining sleep, or early awakening for at least 1 month; (c) patients had no other sleep disorders such as hypersomnia, parasomnia, sleep-related movement disorder, or other psychiatric disorders; (d) patients were younger than 60 years old; (e) free of any psychoactive medication at least 2 weeks prior to and during the study; and (f) patients were right-hand dominant as assessed with the Edinburgh Handedness Inventory. Exclusion criteria were as follows: (a) patients had an abnormal signal in any region of the brain verified by conventional T1-weighted or T2-fluid-attenuated inversion recovery MR imaging; (b) the insomnia disorder was caused by organic disease or severe mental disease such as secondary to depression or generalized anxiety; (c) other sleep disorder; (d) women who were pregnant, nursing, or menstruating; and (e) head motion more than or equal to 1.5 mm or 1.5° during MR imaging. Then three patients were discarded. Finally, 50 PI patients who met the requirements were included in the study.

A total of 40 age-, gender-, and education-matched healthy control (HC) subjects were recruited (17 men, 23 women; mean age, 39.38 ± 9.26 years) from the local community by using advertisements. Each HC subject gave written informed consent. HCs must met the following criterion: (a) Insomnia Severity Index (ISI) score was less than 7; (b) no history of swing shifts, shift work, or sleep complaints; (c) no medication or substance abuse such as caffeine, nicotine, or alcohol; (d) no brain lesions or prior substantial head trauma, which was verified by conventional T1-weighted or T2-fluid-attenuated inversion recovery MR imaging; (e) no history of psychiatric or neurological diseases; (f) head motion less than 1.5 mm or 1.5° during MR scan; and (g) right-hand dominant. Three controls were discarded due to head motion.

### Sleep and Emotion Scales

Several questionnaires were filled out by study participants. These questionnaires included the ISI (39), the Pittsburgh Sleep Quality Index (PSQI) (40), the Self-rating Anxiety Scale (SAS) (41), and the Self-rating Depression Scale (SDS) (42).

### MR Imaging

Resting-state functional MR imaging data were acquired using a 1.5 T MR scanner (Achieva Nova-Dual; Philips, Best, the Netherlands) in the Department of Medical Imaging, Guangdong Second Provincial General Hospital. To minimize head movements, a belt and foam pads were used. During the scanning, subjects were instructed to rest with their eyes closed and remain still but emphatically without falling asleep. The functional MR images were acquired in about 10 min using a gradient-echo planar imaging sequence as follows: interleaved scanning, repetition time = 2,500 ms, echo time = 50 ms, matrix = 64 × 64, field of view = 224 mm × 224 mm, flip angle = 90°, section thickness = 4 mm, gap = 0.8 mm, 27 axial slices, and 240 volumes.

### Data Preprocessing

The Data Processing Assistant for Resting-State Functional MR Imaging toolbox^1^ (version 2.3) was used to process the resting-state functional MR imaging data. Volumes at the first 10 time points were discarded so that magnetization reached a steady state and subjects had adapted to the MR scanning noise. The slice timing and realignment for head motion correction were conducted on the remaining images. Then, the realigned images were spatially normalized to the Montreal Neurological Institute template by applying the EPI template, and each voxel was resampled to 3 mm × 3 mm × 3 mm. We spatially smoothed the spatially normalized images with a 6-mm full-width half-maximum isotropic Gaussian kernel. In order to reduce effects of low-frequency drift and high-frequency noise, we processed the data to remove linear trends and filtered temporally (band-pass, 0.01–0.08 Hz). Nine nuisance covariates, including cerebrospinal fluid signals, white matter signals, global brain signal, and six head motion parameters were regressed from the imaging data. The residuals of these regressions were used for the following analysis.

### Granger Causality Analysis

We calculated the effective connectivity of the time series of the dorsal and ventral rAI on every voxel in the whole brain (*X* to *Y*) and the effective connectivity of the time series of every voxel in the whole brain on the dorsal and ventral rAI (*Y* to *X*). The mean temporal-domain bold signals for the dorsal and ventral rAI are displayed in Figure S10 in Supplementary Material. Regions of interest (ROI) in the dorsal and ventral rAI were selected based on the brain atlas based on connectional architecture (43).^2^ Bivariate first-order coefficient-based voxel-wise Granger causality analysis was performed using REST-GCA (44). We followed Chen’s (37) extended model as following:

$$Y_{t}=\sum_{i=1}^{p}A_{i}X_{\left(t-i\right)}+\sum_{i=1}^{p}B_{i}Y_{\left(t-i\right)}+CZ_{t}+\varepsilon_{t}$$

$$X_{t}=\sum_{i=1}^{p}A_{i}^{\prime }Y_{\left(t-i\right)}+\sum_{i=1}^{p}B_{i}^{\prime }X_{t-i}+C^{\prime }Z_{t}+\varepsilon_{t}^{\prime }$$

where *Y_t_* is the BOLD time series of one voxel in the brain at time *t*; *X* is the BOLD time series of seed region; *Z_t_* is a *q* × 1 vector containing exogenous variables (covariates or confounds) at time *t*; ε*_t_* is the error term; *p* and *q* are the number of lags and confounds, respectively; *A_i_* is the signed path coefficient at time lag *i* (*i* = 1, …, *p*); *B_i_* is the autoregression coefficient. In the present study, the number of lags *p* = 1 (1 TR = 2.5 s).

Explanation of the coefficient was the same as the previous study (38). The positive coefficient is referred as excitatory influence and *vice visa*.

### Statistical Analysis

Differences in age, education level, ISI, PSQI, SAS, and SDS scores between PI patients and HCs were compared by using two-sample *t* tests. Differences associated with gender between the two groups were assessed by using chi-squared tests.

First, the effective connectivity maps were analyzed using one-sample *t*-test for the entire sample (both PI patients and HCs) with an uncorrected *P* < 0.001, cluster size = 50. Then, between-group differences in effective connectivity were compared by using two-sample *t* tests in a voxel-by-voxel fashion with age, sex, and education level imported as covariates. Multiple comparisons were corrected by an AlphaSim method implemented in the DPABI software [DPABI version 2.3, Data Processing & Analysis for (Resting-State) Brain Imaging] (45) and using significant corrected thresholds of *P* < 0.01 with combined with single voxel *P* < 0.001. The estimated FWHM (*x*–*y*–*z*) for the 4T maps (from ventral rAI, from dorsal rAI, to ventral rAI, and to dorsal rAI) were 6.9857–7.1202–7.9652, 5.8353–5.9840–6.7710, 9.1891–9.4015–9.4269, and 5.0546–5.1626–5.8403. The cluster size thresholds for the 4T maps were 22, 16, 48, and 26.

Besides, we used permutation threshold-free cluster enhancement (TFCE) correction method to perform statistical analysis (46, 47). The permutation TFCE correction method implements correction through a permutation testing approach which controls family-wise error rate by comparing voxel-wise statistics (TFCE) to the maximal statistics obtained from repeating the analysis with randomized data. The Matlab scripts for the permutation TFCE correction have been made available online: https://github.com/markallenthornton. ROI were defined as 6-mm-diameter spheres centered on voxels that exhibited the largest absolute *t* value in each of the significant clusters in the *t* map of between-group differences in effective connectivity. Then, effective connectivity was calculated for each subject by averaging the values of effective connectivity across all voxels within each of the ROI and correlated with the sleep and emotion scales using Pearson’s correlation analysis.

## Results

### Sleep and Emotion Scales

As Table 1 shown, the PI patients and the controls showed no significant differences in age (*P* = 0.37), sex (*P* = 0.81), and education level (*P* = 0.28). PI patients had higher ISI, PSQI, SAS, and SDS scores than those of HCs (all *P* < 0.001).

**Table 1:** Demographic, sleep, and emotional scales of all participants.

Variable	PI group (*n* = 50)	HC group (*n* = 40)	*P* value
Sex (M/F)	20/30	17/23	0.81^a^
Age (years)	40.06 ± 8.52	39.38 ± 9.26	0.37^b^
Duration (months)	40.31 ± 44.09	N/A	N/A
Education (years)	7.56 ± 3.24	8.32 ± 3.43	0.28^b^
PSQI	12.55 ± 2.95	5.68 ± 2.46	<0.001^b^
ISI	19.44 ± 3.18	5.78 ± 2.34	<0.001^b^
SAS	51.83 ± 9.27	41.69 ± 5.61	<0.001^b^
SDS	56.03 ± 7.83	42.75 ± 2.64	<0.001^b^

*Unless otherwise noted, data are mean ± SD*.

*PSQI, Pittsburgh Sleep Quality Index; ISI, Insomnia Severity Index; SAS, Self-rating Anxiety Scale; SDS, Self-rating Depression Scale; PI, primary insomnia*.

*^a^The P value was obtained by using chi-square test*.

*^b^The P value was obtained by using two-sample t tests*.

### Effective Connectivity

One-sample *t*-test showed that the rAI exerted excitatory influence on the bilateral dorsolateral prefrontal cortex (DLPFC), the inferior parietal regions, the cingulate gyrus, and the left cerebellar crus. Inhibitory influence of the rAI was noted on the left precentral gyrus, the postcentral gyrus, and the bilateral occipital lobe. Furthermore, the bilateral DLPFC, the inferior parietal regions, and the cingulate gyrus, in turn, had inhibitory influence on the rAI, and in the same way, the bilateral occipital lobe had excitatory influence on the rAI. It is worth noting that the results of one-sample *t*-test were very similar to those of previous study (38). The results of the one-sample *t* tests are presented in Figures S4–S7 in Supplementary Material.

**Figure 1:**
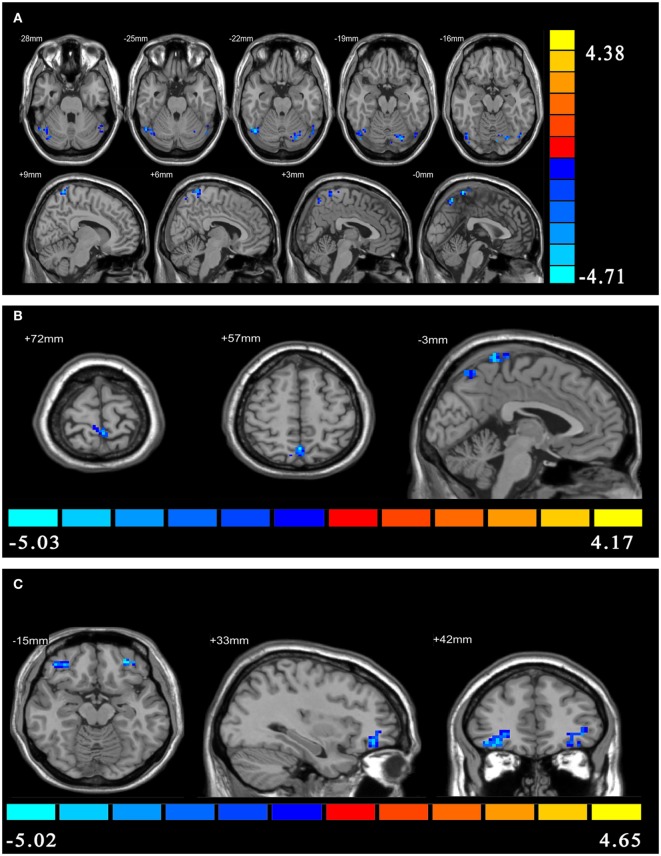
The between-group differences in effective connectivity. **(A)** The between-group differences in effective connectivity from ventral right anterior insula (rAI) to the whole brain. **(B)** The between-group differences in effective connectivity from dorsal rAI to the whole brain. **(C)** The between-group differences in effective connectivity from the whole brain to ventral rAI.

Compared with HCs, patients with PI showed negative effective connectivity (inhibitory influences) from the ventral rAI to the left precuneus, the left postcentral gyrus extending to the bilateral precuneus, and bilateral cerebellum posterior lobe including the bilateral cerebelum_crus1 and left cerebelum_6 (Figure 1A), and negative effective connectivity from the dorsal rAI to the bilateral precuneus and left postcentral gyrus extending to the left precuneus (Figure 1B). Also, patients with PI showed negative effective connectivity from bilateral orbitofrontal cortex (OFC) to ventral rAI (Figure 1C) (single voxel *P* < 0.001, corrected by AlphaSim correction with cluster *P* < 0.01). All above results of between-group differences in effective connectivity are shown in Table 2. Figures S1–S3 in Supplementary Material showed the bar graphs demonstrating the mean effective connectivity values in the ROI defined as 6-mm-diameter spheres centered on voxels that exhibited the largest absolute *t* value in each of the significant clusters in the *t* map.

**Table 2:** Between-group differences in Granger causal influences.

Brain regions	MNI coordinates (mm)	Cluster size (voxels)	*T* values (peak)	Mean values of causal influences in ROI	
				PI group	HC group
**From ventral rAI to following regions**					
Left precuneus	(0 −63 57)	35	−4.61	−0.21 ± 0.33	0.09 ± 0.35
Left postcentral gyrus (extending to bilateral precuneus)	(−3 −45 72)	68	−4.94	−0.12 ± 0.24	0.26 ± 0.48
Left cerebelum_Crus1	(−48 −72 −15)	34	−4.28	−0.07 ± 0.20	0.09 ± 0.23
Right cerebelum_Crus1	(48 −66 −21)	72	−4.97	−0.17 ± 0.31	0.08 ± 0.24
Left cerebelum_6	(−24 −78 −18)	37	−4.66	−0.08 ± 0.20	0.13 ± 0.21

**From dorsal rAI to following regions**					
Bilateral precuneus	(0 −63 57)	45	−5.35	−0.33 ± 0.45	0.14 ± 0.41
Left postcentral gyrus (extending to left precuneus)	(−3 −45 72)	28	−4.94	−0.13 ± 0.30	0.26 ± 0.43

**From following regions to ventral rAI**					
Left orbitofrontal cortex	(−30 45 −15)	90	−5.34	−0.04 ± 0.04	0.00 ± 0.02
Right orbitofrontal cortex	(33 42 −15)	86	−4.93	−0.04 ± 0.05	0.00 ± 0.02

*ROI, regions of interest, which was defined as 6-mm-diameter spheres centered on voxels that exhibited the largest absolute t value in each of the significant clusters in the t map of between-group differences in Granger causal influences*.

*PI, primary insomnia; HC, healthy control, rAI; right anterior insula*.

Results from permutation TFCE correction (5,000 times permutation, default parameters, FWE corrected, *P* < 0.05) were very similar to those derived from our parameter statistical method (two-sample *t* test with AlphaSim correction). Therefore, we only discussed these results. Figure S8 in Supplementary Material showed the *P* map of the permutation TFCE correction.

### Relationships Between Effective Connectivity and Sleep and Emotion Scales

**Figure 2:**
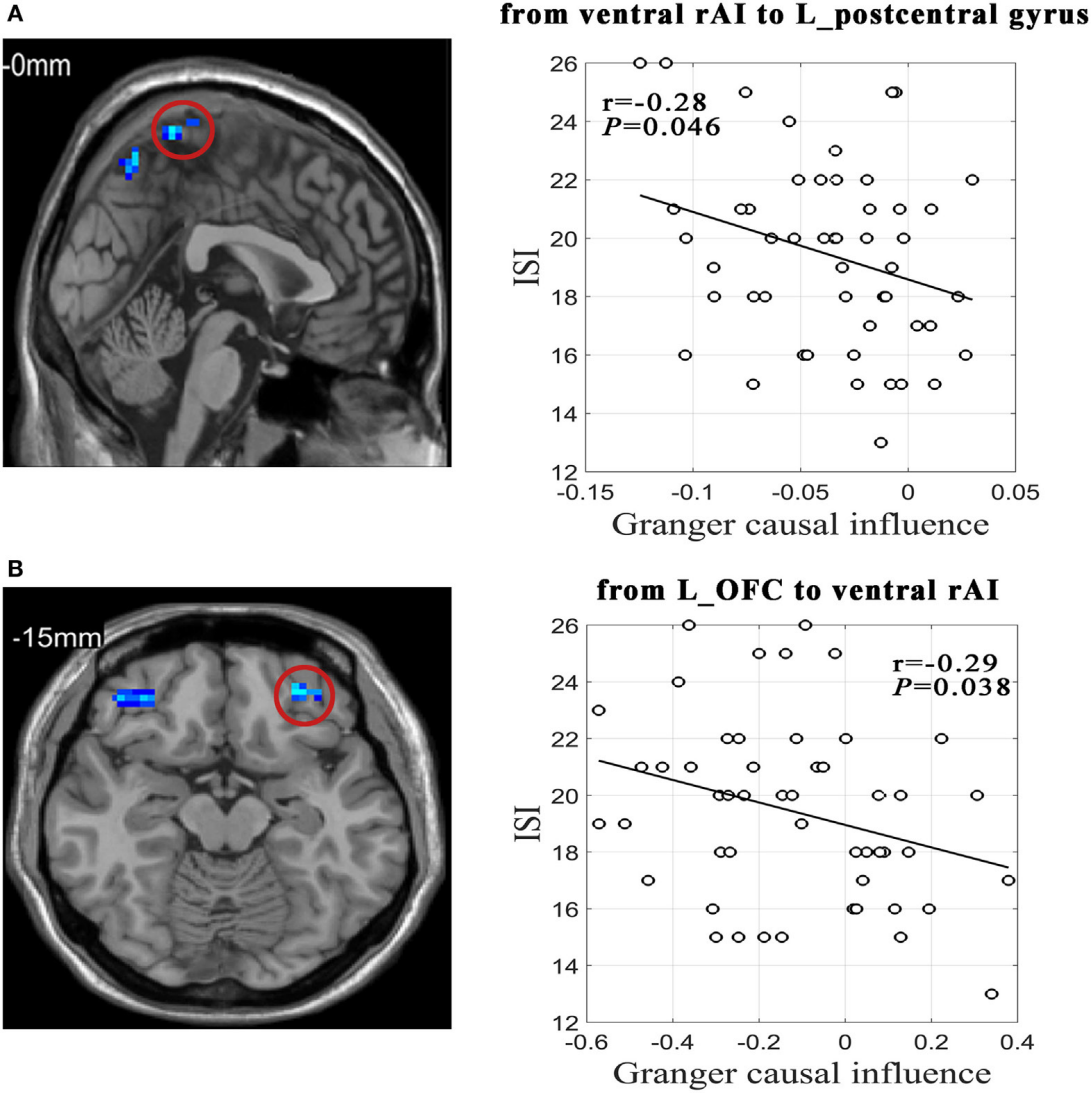
Relationships between effective connectivity and sleep and emotion scales in primary insomnia group. **(A)** Scatter plot shows correlation between effective connectivity value from ventral right anterior insula (rAI) to left postcentral gyrus extending to bilateral precuneus and Insomnia Severity Index (ISI) scores. **(B)** Scatter plot shows correlation between effective connectivity value from left orbitofrontal cortex (L_OFC) to ventral rAI and ISI.

As Figure 2 shown, effective connectivity from the ventral rAI to the left postcentral gyrus extending to the bilateral precuneus and from the left OFC to the ventral rAI were significantly negatively correlated with ISI scores in PI group (*r* = −0.28/*P* = 0.046 and *r* = −0.29/*P* = 0.038, respectively).

Effective connectivity from the ventral rAI to the left precuneus was significantly negatively correlated with PSQI and ISI scores in HC group (*r* = −0.31/*P* = 0.047 and *r* = −0.32/*P* = 0.045, respectively). Figure S9 in Supplementary Material shows the results of the correlation analysis of the HC group.

## Discussion

The present study investigated the effective connectivity between the rAI and the whole brain in PI patients. Our findings showed aberrant effective connectivity of rAI (a key node of SN) with the posterior DMN hub (precuneus) as well as regions involved in decision-making (OFC) and regions involved in sensori-motor function in PI. In addition, effective connectivity from the ventral rAI to the left postcentral gyrus extending to the bilateral precuneus and from the left OFC to the ventral rAI were significantly negatively correlated with ISI scores in PI group.

In contrast to FC which does not support inferences about directional brain connections, effective connectivity refers to the influence that one neural system exerts over another and quantifies the directional connectivity among brain regions (38). Consequently, effective connectivity may provide new insight into the neurological mechanism of insomnia.

The important findings in the current study were aberrant effective connectivity from the ventral rAI to the left precuneus and from the dorsal rAI to the bilateral precuneus at resting state. The rAI was a hub node of SN which is involved in detecting and orienting to both external and internal salient stimuli and events (31, 32). The precuneus was a hub node of DMN which is involved in self-referential/internally oriented processes (48). Previous study using chronometry and Granger causality analysis confirmed that rAI plays a critical and causal role in switching between the ECN and the DMN during visual attention tasks, oddball tasks, and even resting state (30). Furthermore, the present theory holds that one fundamental mechanism underlying cognitive control is a transient signal from the right fronto-insular cortex, which engages the brain’s attentional, working memory, and higher-order control processes while disengaging other systems (such as DMN) that are not immediately task relevant (30, 32). Interestingly, a recent study found that PI patients showed both reduced activation in task-related working memory regions and reduced deactivation in regions of the DMN with increasing task difficulty (15). This finding demonstrated a failed disengagement from DMN during working memory tasks in PI patients. It is a complement to previous studies that only found decreased metabolism or decreased activation in cognitive or task-related regions (13, 14). In our study, although we did not found any altered effective connectivity from rAI to ECN, we found aberrant effective connectivity from the rAI to regions of DMN at resting state or baseline condition. Our findings offer a parsimonious explanation for failed disengagement from DMN during cognitively demanding tasks (especially the working memory task) in PI patients.

Another finding was that PI patients showed aberrant effective connectivity from the bilateral OFC to the ventral rAI. Besides, the effective connectivity from left OFC to ventral rAI was significantly negatively correlated with ISI scores in PI group. The OFC is an important brain area responsible for emotion and decision-making (49). Previous study showed that PI patients’ speed are slower than controls on a vigilance task which only need decision-making (50). Impaired decision-making may also lead to a lack of ability to solve problems in the insomnia patients (51). Indeed, PI patients showed reduced orbitofrontal gray matter volume or density (27, 28). Recent studies found that rAI also acted as a main outflow hub within SN for easier decision-making task (52). Together with previous studies, our findings suggest an abnormal OFC-rAI circuit in PI patients, which might be one of underlying substrate of impaired decision-making observed in PI patients.

We also found that PI patients showed aberrant effective connectivity from the rAI to the left postcentral gyrus (extending to bilateral precuneus) and the bilateral cerebellum posterior lobe. In addition, effective connectivity from ventral rAI to the left postcentral gyrus was significantly negatively correlated with ISI scores in the PI group. The postcentral gyrus and the cerebellum are locations of primary somatosensory cortex and motor control area, respectively. In recent years, several studies have also frequently reported abnormal spontaneous brain activity in the cerebellum posterior lobe as well as the postcentral gyrus in PI (16, 17, 53). On the other hand, existing evidence suggests the anterior insula and the anterior cingulate cortex serve as complementary limbic sensory and motor regions. They work together, similar to the somatosensory and motor cortices (54, 55). Relative to rAI, which is high on the level of the hierarchy due to its function of switching between other large-scale networks, the postcentral gyrus and the cerebellum is lower in the hierarchy (32, 56). Therefore, our findings that aberrant effective connectivity from the rAI to the left postcentral gyrus and the bilateral cerebellum posterior lobe may reflect aberrant top-down sensory and motor control of rAI in PI patients.

Our study had several limitations. First, it was a cross-sectional study, and we cannot directly identify the causal relation between PI and the abnormal effective connectivity. Longitudinal studies may help address this question. Second, we did not directly investigate the inter-network effective connectivity among SN, ECN, and DMN using independent component analysis, even though selected seed regions of rAI for Granger causality analysis was the widely recognized hub node of SN. Future researchers can use independent component analysis to study inter-network effective connectivity in PI. Third, analyses of combination of mental chronometry and Granger causality analysis will increase our understanding of PI. However, we did not do these analyses for technical reasons. Future study is suggested to do these analyses. Finally, the activity of the brain at resting state is not static but is a highly dynamic system. Therefore, static effective connectivity may not be enough to fully characterize the human brain. Future study is suggested to use dynamic FC to investigate the brain in PI.

In summary, we for the first time found aberrant effective connectivity of rAI (a key node of SN) with the posterior DMN hub (precuneus) as well as regions involved in decision-making (OFC) and regions involved in sensori-motor function in PI. These findings suggest an aberrant salience processing system of the rAI, which may be a candidate substrate for cognitive impairment, especially the impairment of working memory and decision-making in PI patients.

## Ethics Statement

This study was carried out in accordance with the recommendations of “ethics committee of the Guangdong Second Provincial General Hospital” with written informed consent from all subjects. All subjects gave written informed consent in accordance with the Declaration of Helsinki. The protocol was approved by the “ethics committee of the Guangdong Second Provincial General Hospital.”

## Author Contributions

Study concepts/study design or data acquisition, manuscript drafting for important intellectual content, and approval of final version of submitted manuscript: all authors; literature research: CL, YY, KH, SF, and GJ; clinical studies: CL, MD, and GJ; experimental studies: CL and GJ; statistical analysis: CL and SF; and manuscript editing: CL, MD, and GJ.

## Conflict of Interest Statement

The authors declare that the research was conducted in the absence of any commercial or financial relationships that could be construed as a potential conflict of interest.

## Footnotes

^1^http://www.restfMRI.net/forum/DPARSF (Accessed: July, 2017).

^2^http://atlas.brainnetome.org/ (Accessed: July, 2017).

## Supplementary Material

Figure S1 The bar graphs demonstrating the mean effective connectivity values in the regions of interest defined as 6-mm-diameter spheres centered on voxels that exhibited the largest absolute *t* value in each of the significant clusters in the *t* map. Error bars indicate SD. Abbreviations: L_CER_crus1, left cerebelum_crus1; R_CER_crus1, right cerebelum_crus1; L_CER_6, left cerebelum_6; L_precuneus, left precuneus; L_postcentral gyrus, left postcentral gyrus (extending to bilateral precuneus).

Figure S2 The bar graphs demonstrating the mean effective connectivity values in the regions of interest defined as 6-mm-diameter spheres centered on voxels that exhibited the largest absolute *t* value in each of the significant clusters in the *t* map. Error bars indicate SD. Abbreviations: Bi_precuneus, bilateral precuneus; L_postcentral gyrus, left postcentral gyrus (extending to left precuneus).

Figure S3 The bar graphs demonstrating the mean effective connectivity values in the regions of interest defined as 6-mm-diameter spheres centered on voxels that exhibited the largest absolute *t* value in each of the significant clusters in the *t* map. Error bars indicate SD. Abbreviations: R_OFC, right orbitofrontal cortex; L_OFC, left orbitofrontal cortex.

Figure S4 The effective connectivity from ventral right anterior insula to the whole brain.

Figure S5 The effective connectivity from dorsal right anterior insula to the whole brain.

Figure S6 The effective connectivity from the whole brain to ventral right anterior insula.

Figure S7 The effective connectivity from the whole brain to dorsal right anterior insula.

Figure S8 The statistical results of the permutation threshold-free cluster enhancement correction (5,000 times permutation, default parameters, FWE corrected, *P* < 0.05). **(A)** Between-group differences in effective connectivity from ventral right anterior insula (rAI) to the whole brain. **(B)** Between-group differences in effective connectivity from dorsal rAI to the whole brain. **(C)** Between-group differences in effective connectivity from the whole brain to ventral right anterior insula.

Figure S9 Relationships between effective connectivity and sleep and emotion scales in healthy control group.

Figure S10 Temporal-domain bold signals for seed regions.
