# Supplementary figures and images for: Aberrant Effective Connectivity of the Right Anterior Insula in Primary Insomnia
Source: Front Neurol. 2018 May 8;9:317. doi: 10.3389/fneur.2018.00317 (PMC5951943; doi:10.3389/fneur.2018.00317)

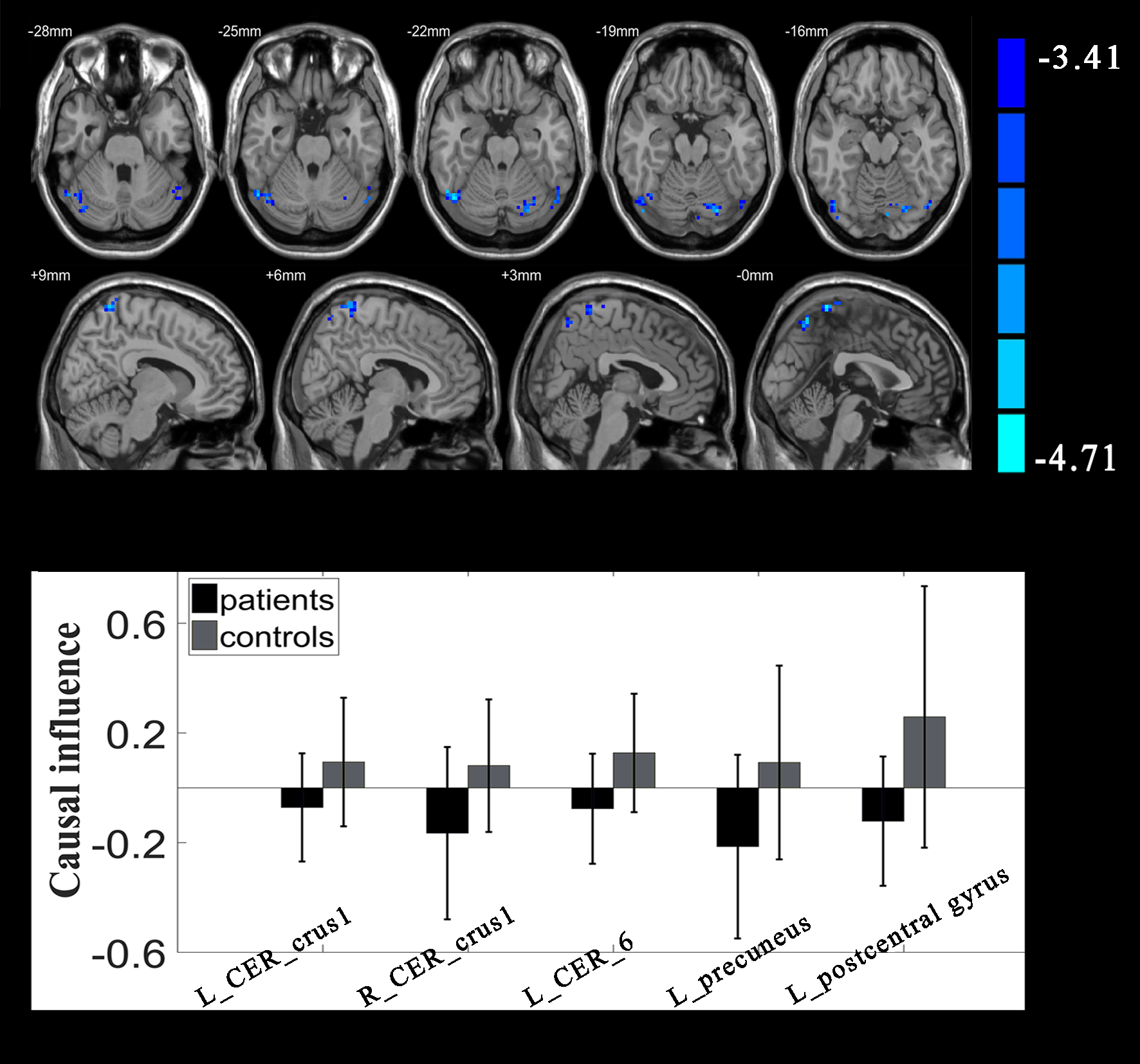

Supplement: Figure S1 — The bar graphs demonstrating the mean effective connectivity values in the regions of interest defined as 6-mm-diameter spheres centered on voxels that exhibited the largest absolute t value in each of the significant clusters in the t map. Error bars indicate SD. Abbreviations: L_CER_crus1, left cerebelum_crus1; R_CER_crus1, right cerebelum_crus1; L_CER_6, left cerebelum_6; L_precuneus, left precuneus; L_postcentral gyrus, left postcentral gyrus (extending to bilateral precuneus). [file Image_1.tif]

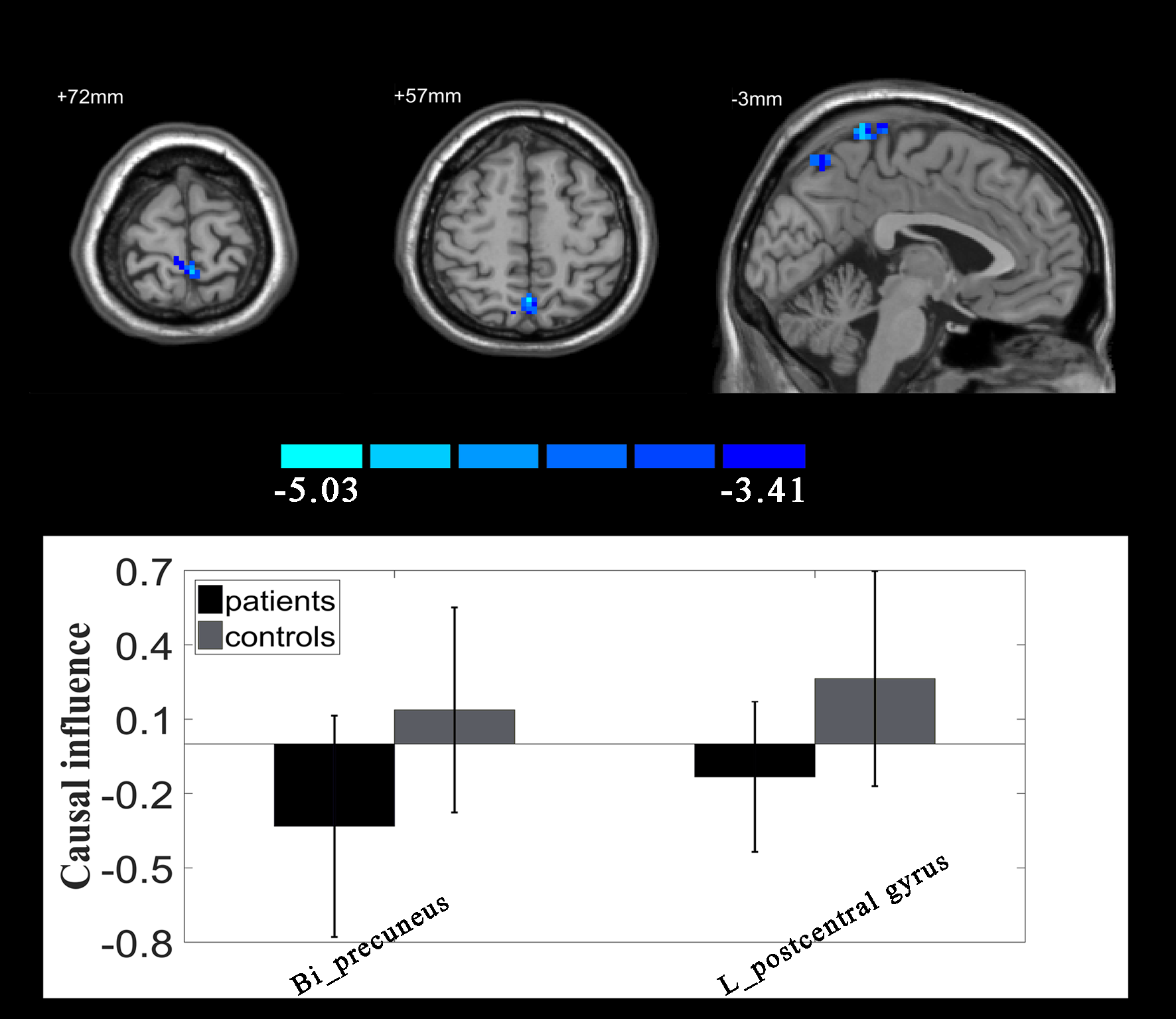

Supplement: Figure S2 — The bar graphs demonstrating the mean effective connectivity values in the regions of interest defined as 6-mm-diameter spheres centered on voxels that exhibited the largest absolute t value in each of the significant clusters in the t map. Error bars indicate SD. Abbreviations: Bi_precuneus, bilateral precuneus; L_postcentral gyrus, left postcentral gyrus (extending to left precuneus). [file Image_2.tif]

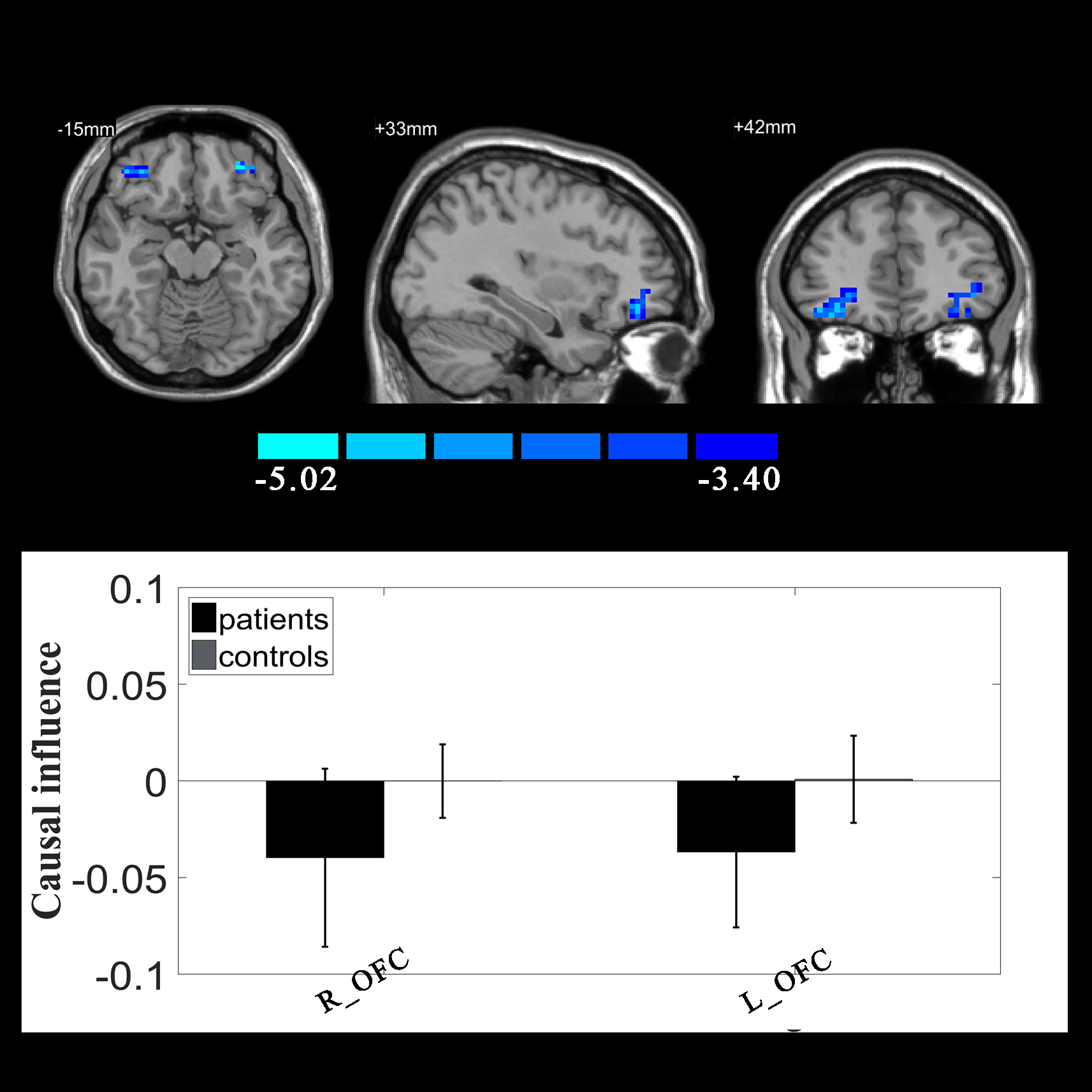

Supplement: Figure S3 — The bar graphs demonstrating the mean effective connectivity values in the regions of interest defined as 6-mm-diameter spheres centered on voxels that exhibited the largest absolute t value in each of the significant clusters in the t map. Error bars indicate SD. Abbreviations: R_OFC, right orbitofrontal cortex; L_OFC, left orbitofrontal cortex. [file Image_3.tif]

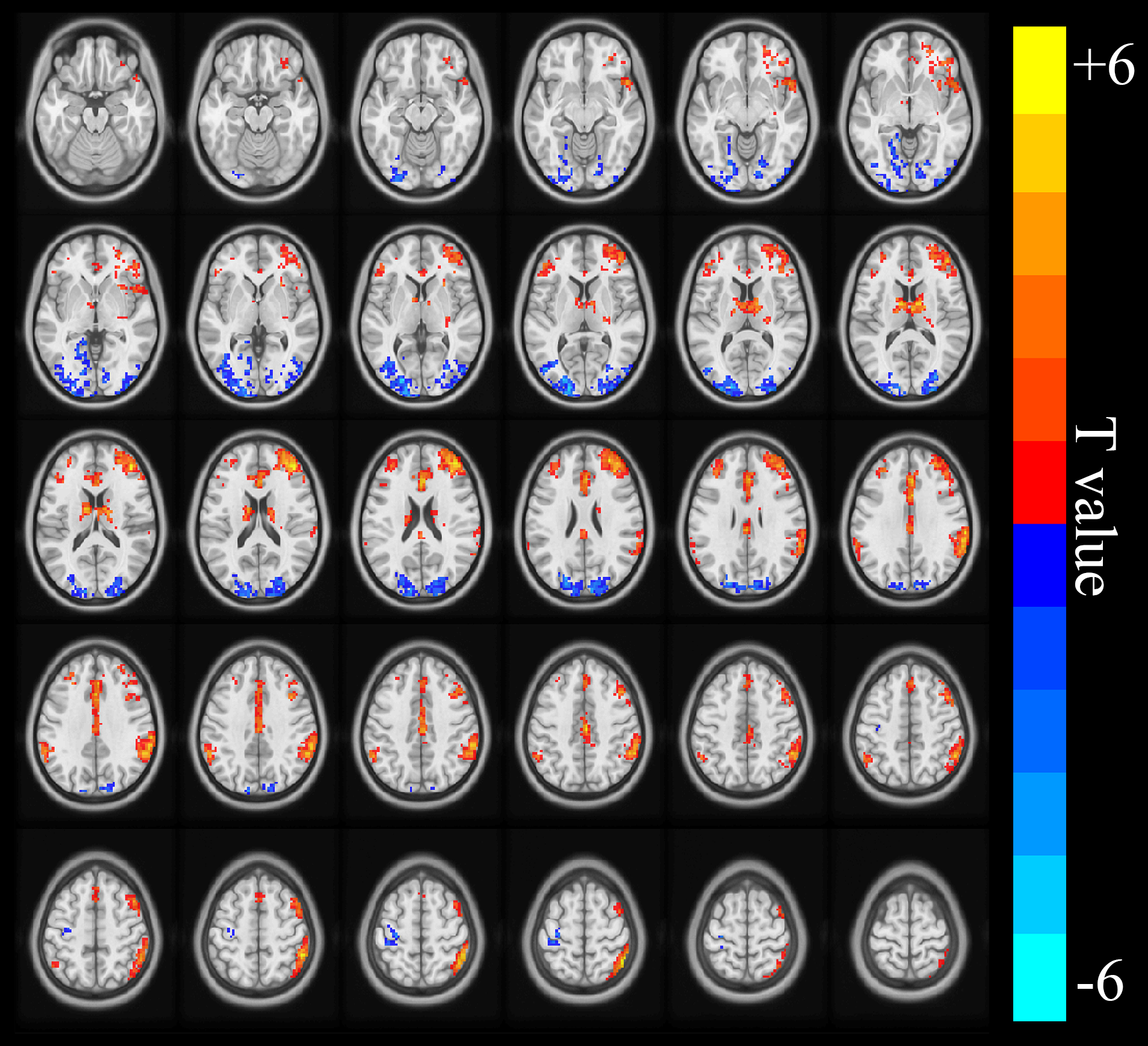

Supplement: Figure S4 — The effective connectivity from ventral right anterior insula to the whole brain. [file Image_4.tif]

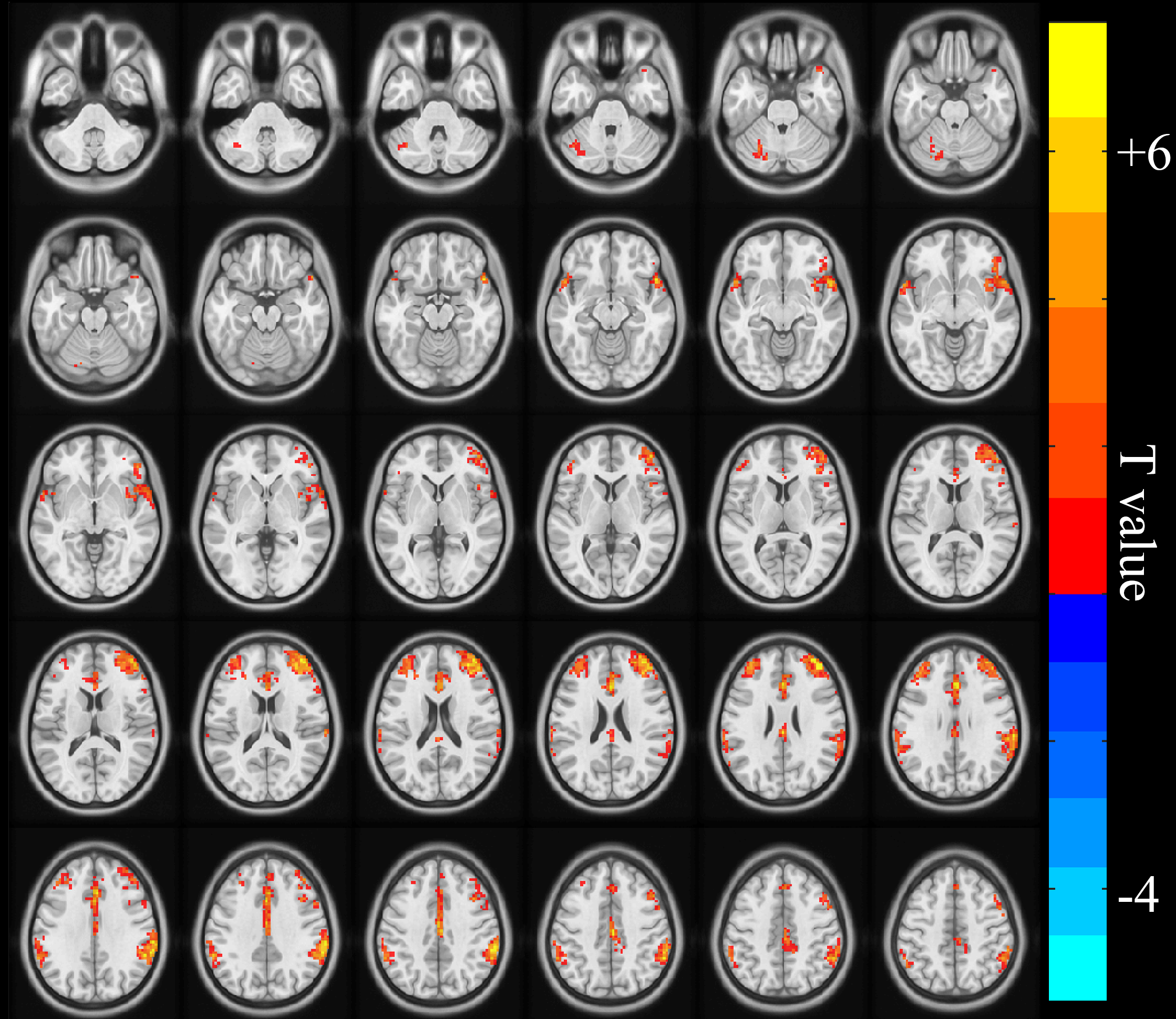

Supplement: Figure S5 — The effective connectivity from dorsal right anterior insula to the whole brain. [file Image_5.tif]

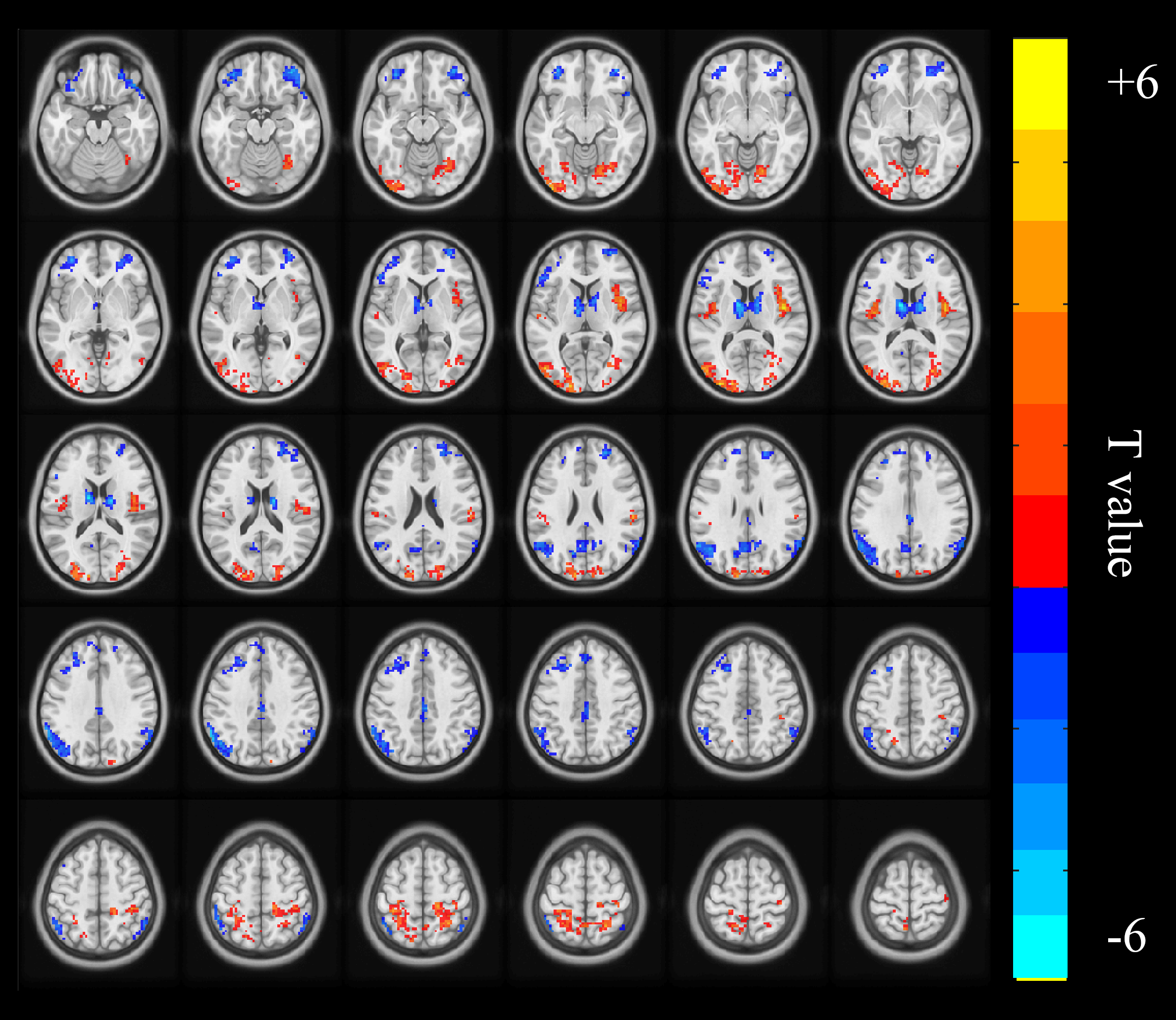

Supplement: Figure S6 — The effective connectivity from the whole brain to ventral right anterior insula. [file Image_6.tif]

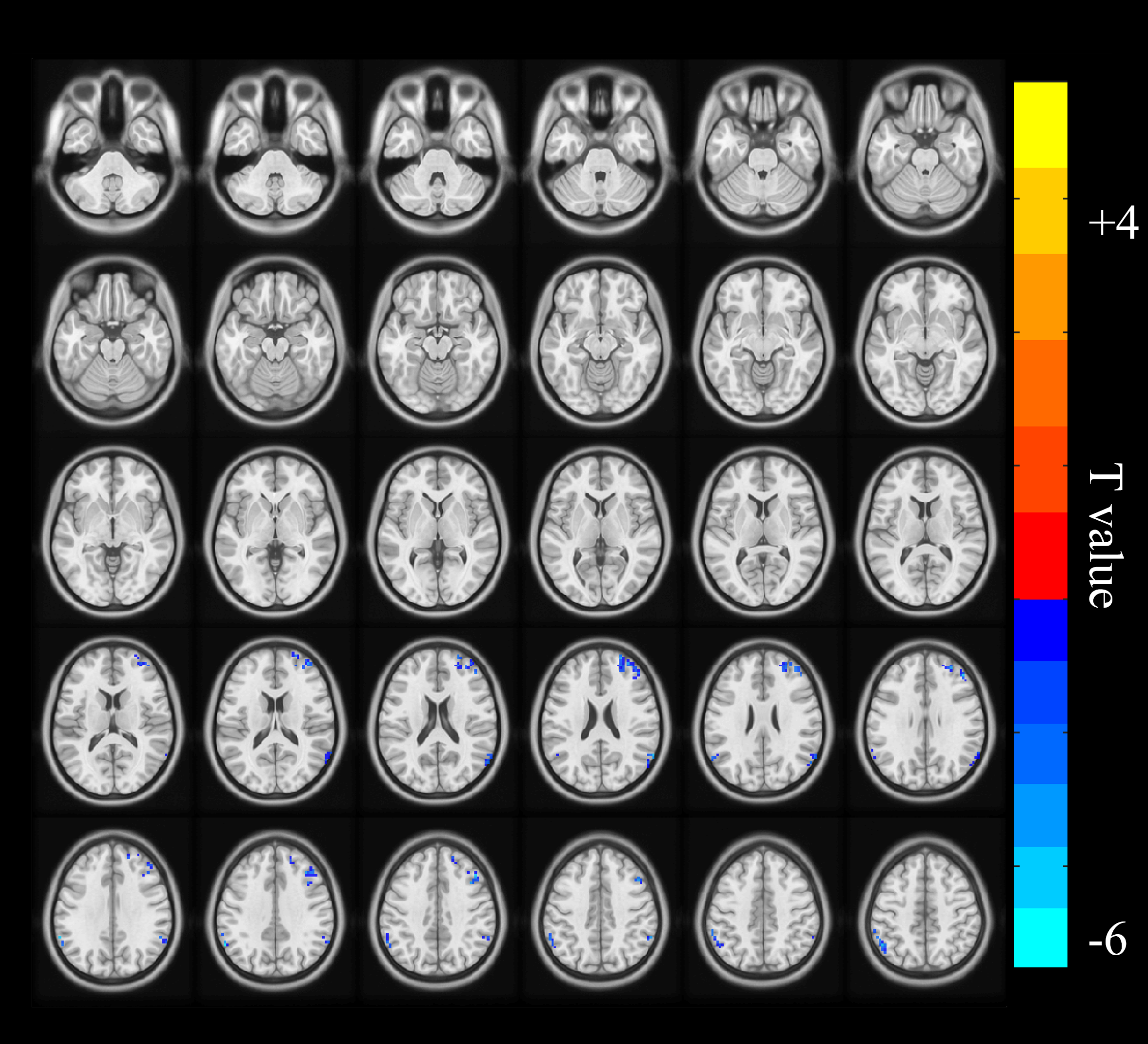

Supplement: Figure S7 — The effective connectivity from the whole brain to dorsal right anterior insula. [file Image_7.tif]

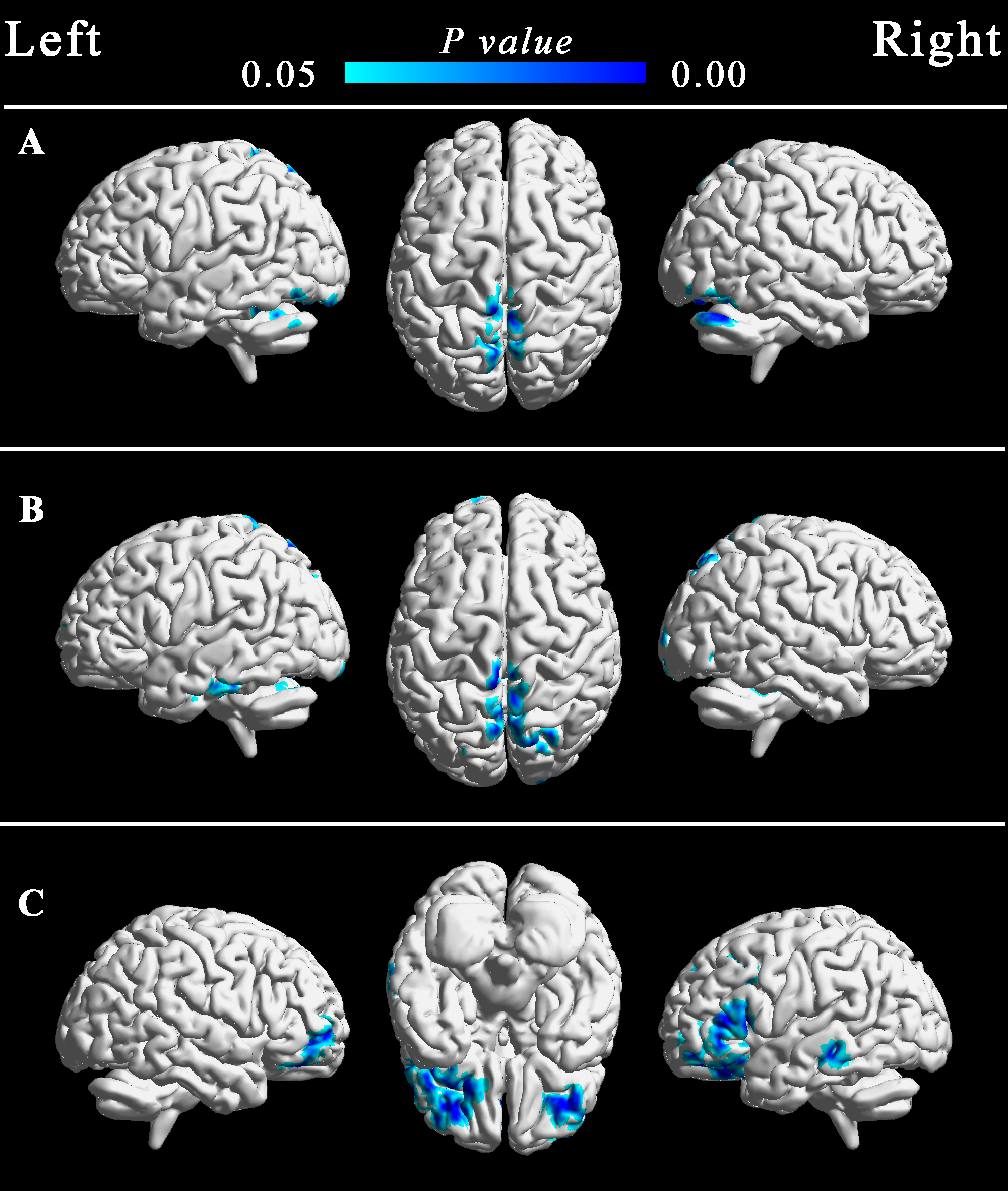

Supplement: Figure S8 — The statistical results of the permutation threshold-free cluster enhancement correction (5,000 times permutation, default parameters, FWE corrected, P < 0.05). (A) Between-group differences in effective connectivity from ventral right anterior insula (rAI) to the whole brain. (B) Between-group differences in effective connectivity from dorsal rAI to the whole brain. (C) Between-group differences in effective connectivity from the whole brain to ventral right anterior insula. [file Image_8.tif]

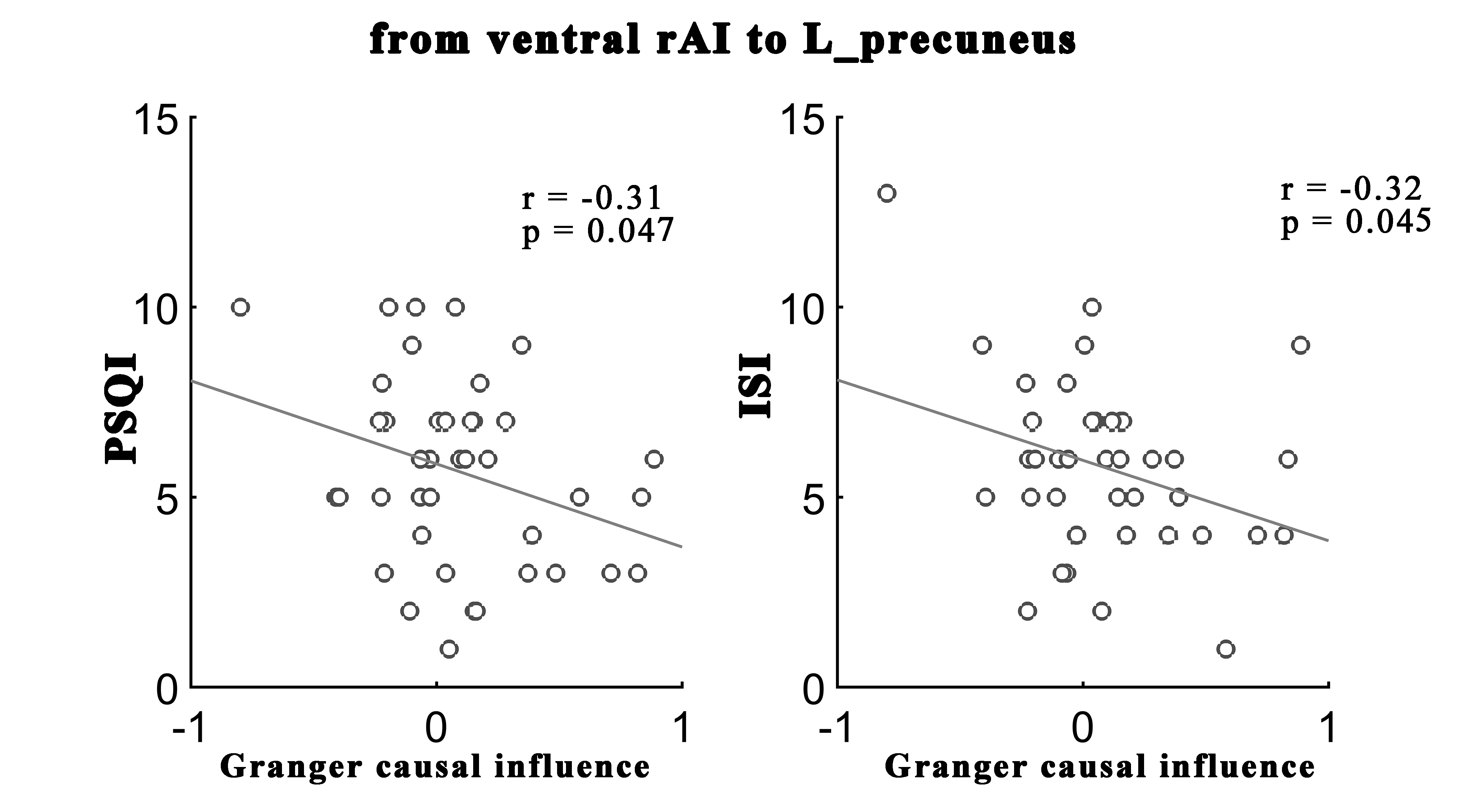

Supplement: Figure S9 — Relationships between effective connectivity and sleep and emotion scales in healthy control group. [file Image_9.tif]

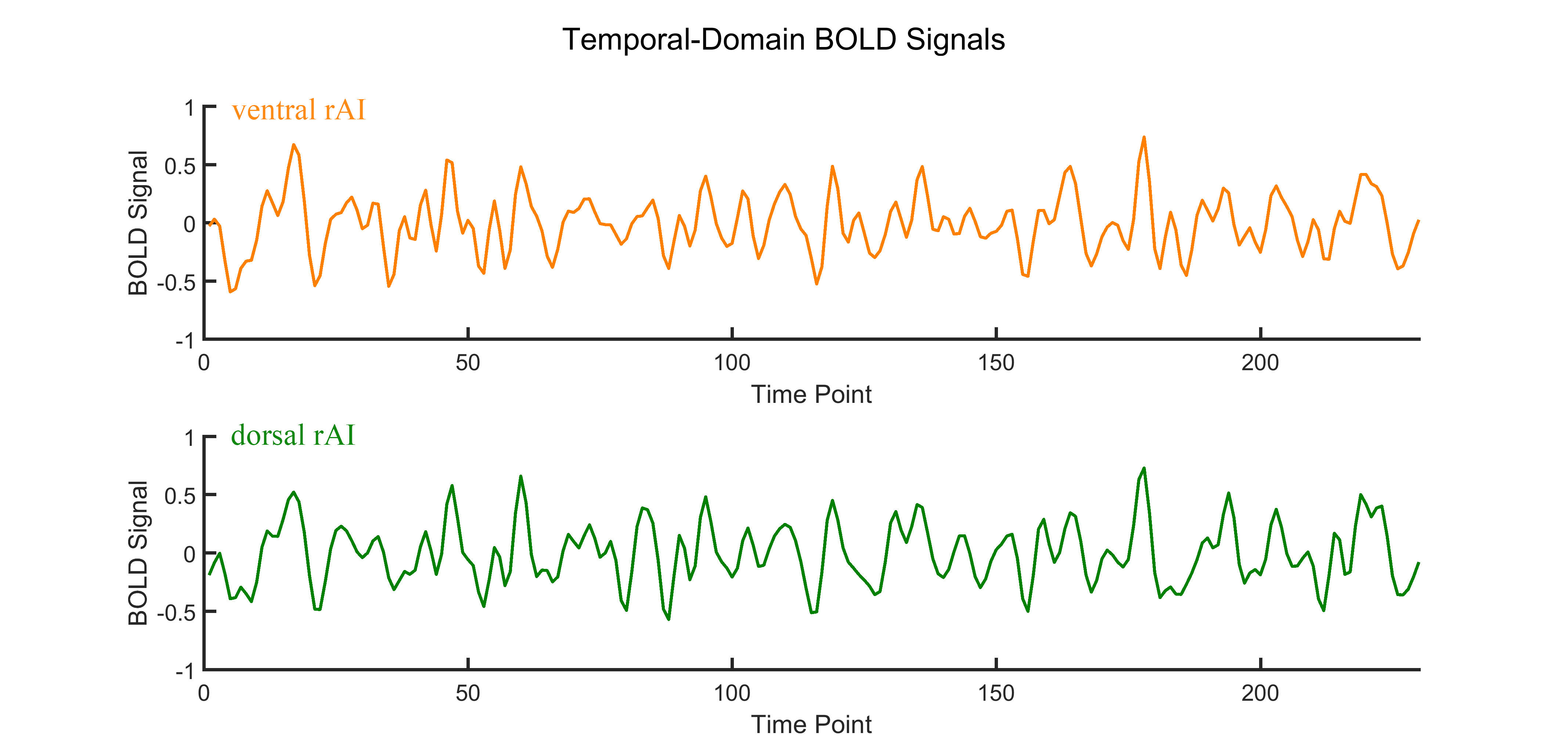

Supplement: Figure S10 — Temporal-domain bold signals for seed regions. [file Image_10.tif]
